# Smaller self-inflating bags produce greater guideline consistent ventilation in simulated cardiopulmonary resuscitation

**DOI:** 10.1186/1471-227X-9-4

**Published:** 2009-02-20

**Authors:** Ziad Nehme, Malcolm J Boyle

**Affiliations:** 1 Monash University, Department of Community Emergency Health and Paramedic Practice, PO Box 527, Frankston 3199, Victoria, Australia; 2 Ambulance Victoria, PO Box 2000, Doncaster 3108, Victoria, Australia

## Abstract

**Background:**

Suboptimal bag ventilation in cardiopulmonary resuscitation (CPR) has demonstrated detrimental physiological outcomes for cardiac arrest patients. In light of recent guideline changes for resuscitation, there is a need to identify the efficacy of bag ventilation by prehospital care providers. The objective of this study was to evaluate bag ventilation in relation to operator ability to achieve guideline consistent ventilation rate, tidal volume and minute volume when using two different capacity self-inflating bags in an undergraduate paramedic cohort.

**Methods:**

An experimental study using a mechanical lung model and a simulated adult cardiac arrest to assess the ventilation ability of third year Monash University undergraduate paramedic students. Participants were instructed to ventilate using 1600 ml and 1000 ml bags for a length of two minutes at the correct rate and tidal volume for a patient undergoing CPR with an advanced airway. Ventilation rate and tidal volume were recorded using an analogue scale with mean values calculated. Ethics approval was granted.

**Results:**

Suboptimal ventilation with the use of conventional 1600 ml bag was common, with 77% and 97% of participants unable to achieve guideline consistent ventilation rates and tidal volumes respectively. Reduced levels of suboptimal ventilation arouse from the use of the smaller bag with a 27% reduction in suboptimal tidal volumes (p = 0.015) and 23% reduction in suboptimal minute volumes (p = 0.045).

**Conclusion:**

Smaller self-inflating bags reduce the incidence of suboptimal tidal volumes and minute volumes and produce greater guideline consistent results for cardiac arrest patients.

## Background

The ability to deliver adequate ventilation via a self-inflating bag in the presence or absence of an advanced airway is a skill that requires comprehensive initial training and ongoing assessment and reinforcement. In recent times, the literature has identified a lack of compliance with ventilation guidelines by emergency care providers in the field, with much of the research highlighting an association between overzealous ventilation and poorer outcomes in cardiac arrest [1], hypovolaemic shock [2] and severe head injury [3]. In light of this evidence and changes to the International Liaison Committee on Resuscitation (ILCOR) guidelines for resuscitation, there is a need to investigate and observe the efficacy of manual ventilation among prehospital care providers in relation to operator delivery of ventilation rate and tidal volume[4]

There is no literature describing the ability of undergraduate paramedic students to accurately ventilate, using a self-inflating bag, in a simulated adult cardiac arrest patient. Previous international studies involving prehospital care providers have demonstrated poor compliance with recommended ventilation guidelines. [5-9] Furthermore, there is no Australian context relating the ability of infield paramedics to successfully ventilate an apnoeic or hypoventilating patient.

The objective of this study was to evaluate bag ventilation in relation to operator ability to achieve guideline consistent ventilation rate, tidal volume and minute volume when using two different capacity self-inflating bags in an undergraduate paramedic cohort.

## Methods

### Study Design

An experimental study using a mechanical lung model to determine ventilation rate, tidal volume and minute volume in a simulated adult cardiac arrest scenario.

### Population and Setting

Undergraduate paramedic students in the third year of a pre-registration course, Bachelor of Emergency Health (Paramedic) at Monash University, Victoria, Australia were eligible for inclusion in the study. There were 70 students eligible for inclusion in the study, with a convenience sample of third year students used in the study. At the time of enrolment, participants had undertaken over 28 months (or equivalent prior learning) of clinical education at Monash University while a clinical placement program ensured that each participant had undertaken at least 300 hours of in-field practice. While participants were in the process of completing their final year of study, the theory and practice relating to CPR were established in prior subjects of the course. Students were expected to understand and practice according to the 2005 ILCOR resuscitation guidelines. There were no exclusion criteria.

### Process

A full-torso manikin (Resusci Anne Simulator, Laerdal, Victoria, Australia) was used to represent a simulated 80 kg adult cardiac arrest patient. Ventilation rate, tidal volume and minute volume were measured using a mechanical lung model (Training/Test Lung Model 1601, Michigan Instruments Inc., Michigan, U.S.A) with a lung compliance and airway resistance values set at 0.05 L/cmH_2_O and 5 cmH_2_0/L/sec respectively. Participants were instructed to ventilate using 1600 ml (Adult Silicone Resuscitator, Laerdal, Victoria, Australia) and 1000 ml (Paediatric Resuscitator AF2040, VentLab Corp., North Carolina, USA) bags using two hands for a length of two minutes for a patient undergoing CPR with an advanced airway. The starting bag was selected randomly using a random numbers table. Participants were rated on their ability to achieve guideline consistent ventilation as described by the ILCOR resuscitation guidelines[4] The following individual measures were considered guideline consistent:

1. a ventilation rate between 8 and 10, inclusive;

2. a tidal volume between 480 ml and 560 ml inclusive (based on 6–7 ml/kg for the 80 kg simulated patient); and

3. a minute volume between 3840 ml and 5600 ml inclusive (based on multiple of lowest and highest acceptable ventilation rate and tidal volume).

Ventilation rate and tidal volume were recorded by one of the researchers (ZN) directly from the mechanical lung model using an analogue scale. The term "suboptimal ventilation" was attributed to any mean value that did not fall within the range of accepted ILCOR resuscitation guidelines.

### Analysis

Ventilation rate and tidal volumes were calculated as mean values over a two minute period. Descriptive statistics were used to describe the demographics of the study sample, measures of central tendency were used to summate the results of each bag size, and the student's *t*-test was used to compare the two bag sizes. Statistical analysis was undertaken using SPSS 15.0 (Statistical Package for the Social Sciences Version 15.0, SPSS Inc., Chicago, Illinois, U.S.A). All confidence intervals (CI) are 95%, results were considered statistically significant if the p-value was less than 0.05.

### Ethics

Ethics approval was granted by the Monash University's Standing Committee on Ethics in Research involving Humans (SCERH).

## Results

A total of 30 undergraduate paramedic students (15 male, 15 female) participated in the study. Over half of all participants were aged between 21–25 years (n = 17), while the next most common age group was 26–30 years with 9 participants. Age groups 31–35, 36–40 and 41–45 were attributed to population numbers of 1, 2 and 1 respectively. There were no participants aged below 21 years or over 45 years.

**Figure 1:**
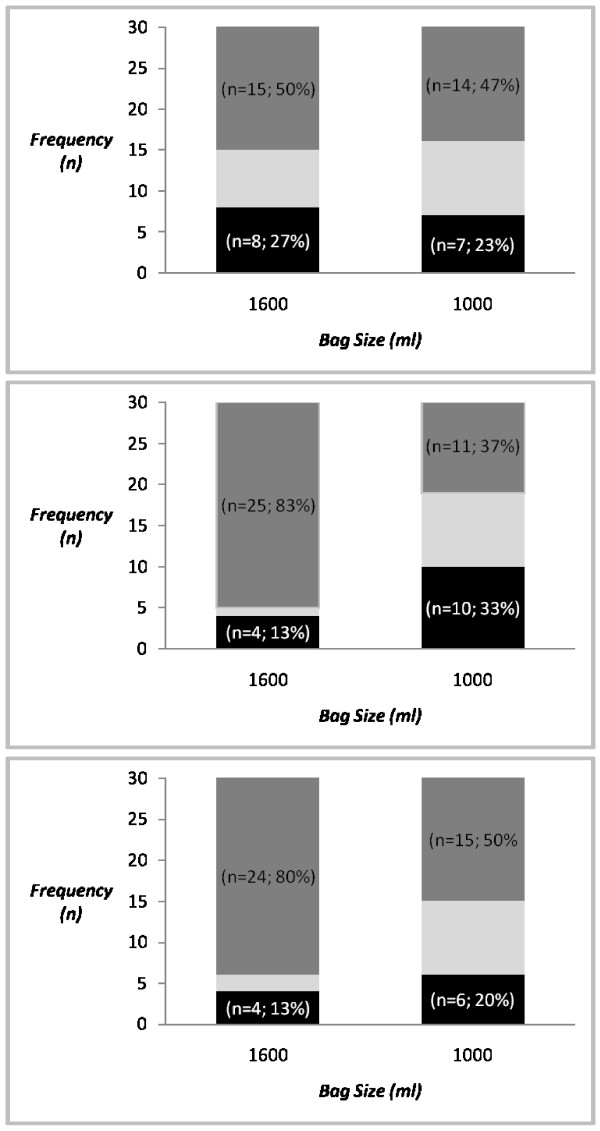
**Suboptimal Ventilation**. a) Frequency of suboptimal ventilation rates (< 8 VPM or > 10 VPM). b) Frequency of suboptimal tidal volumes (< 480 ml or > 560 ml). c) Frequency of suboptimal minute volumes (< 3840 ml or > 5600 ml). Black shade: denotes hypoventilation; Light shade: denotes guideline consistent ventilation; Dark shade: denotes hyperventilation. All measurements are based on mean values calculated over the two minute period.

The analysis found that 77% of participants (n = 23) who used the 1600 ml bag, and 70% of participants (n = 21) who used the 1000 ml bag were suboptimal with their ventilation rates (Figure 1a). While there appeared to be a trend for greater guideline consistent results in relation to ventilation rate when using the 1000 ml bag, a statistically significant result was not observed (p = 0.770).

Assessment of tidal volumes revealed that 97% of participants (n = 29) were unable to achieve guideline consistent tidal volumes when undertaking ventilation in the simulated patient using the 1600 ml self-inflating bag. We found that participants using the smaller 1000 ml bag were more likely to achieve optimal tidal volumes in the simulated patient (p = 0.015). Seventy percent (n = 21) of participants produced mean tidal volumes consistent with either hypoventilation or hyperventilation of the simulated patient. Of the participants who used the 1000 ml bag, 70% (n = 21) participants were also suboptimal with their tidal volumes (Figure 1b).

Similarly, mean minute volumes over the two minute period revealed significant differences between the two capacity bags. We found that participants who used the smaller self-inflating bag were more likely to achieve guideline consistent minute volumes. Ninety-three percent of participants (n = 28) using the 1600 ml bag and 70% (n = 21) of participants using the 1000 ml bag demonstrated suboptimal minute volumes for the simulated patient (p = 0.045), see Figure 1.

No statistically significant difference was found when comparing gender-specific performance in relation to ventilation rate, tidal volume or minute volume.

## Discussion

Ventilation of a simulated adult cardiac arrest patient by undergraduate Monash University paramedic students is better achieved by using a smaller self-inflating bag. Even when using the smaller self-inflating bag ventilation values were still predominately suboptimal.

It is now well supported that the delivery of ventilation using a self-inflating bag is erratic and uncontrolled by all disciplines, not just prehospital care providers[6,10-12,1] Pitts and Kellermann proposed that hyperventilation is inevitable in real life situations and is perhaps not rescuer training that requires re-visiting, rather controlling operator emotions at the time of the incident... *"When the alarm sounds, we rush to the scene uncertain of what we will find. The suddenness of these events and the high stakes involved produces an adrenaline-driven arousal response. As a result, we do everything fast, including, perhaps, bag-valve ventilation." *[13] In an effort to exclude the emotion that is often associated with real-life circumstances, this study explored the degree of suboptimal ventilation within the control of a simulated clinical scenario.

We found that 77% (n = 23) of participants who used the 1600 ml bag and 70% (n = 21) of participants using the smaller bag were unable to reach the target ventilation rate of 8–10 VPM. Other authors have documented similar trends in ventilation rates during CPR. Aufderheide and colleagues found mean ventilation rates to be as high as 30 VPM in 7 men undergoing CPR with an advanced airway,[1] while other authors have observed rates as high as 70 VPM by some emergency care providers [14]. In a more recent study, emergency department personnel were observed to have ventilated 12 cardiac arrest patients at a median rate of 21 VPM[15]

Based on ILCOR's recommendation of 6 – 7 ml/kg, we observed that suboptimal tidal volumes could be reduced by 27% if operators used a smaller 1000 ml capacity bag. Ninety-seven percent (n = 29) of all participants were unable to ventilate within the recommended tidal volume for the simulated patient when using the conventional 1600 ml bag – a potentially catastrophic outcome for cardiac arrest patients in the field. A similar result was found with minute volumes, with the level of suboptimal ventilation reducing from 93% in participants using the 1600 ml bag to 70% (n = 21) in participants using the smaller 1000 ml bag (p = 0.045).

Doerges and colleagues were one of the first to query the difficulty in reaching new ventilation targets with current capacity adult bags. Their study found that ventilation using an adult capacity bag via an advanced airway usually resulted in tidal volumes as high as 1000 ml and often over-shooting the recommended 400–600 ml by the ERC[16] Mean minute volumes of almost 20 litres were also noted with the use of a large bag. When compared to a paediatric 700 ml bag, they found that they were able to reduce tidal volumes to a mean of 389 ml ± 113 and therefore significantly reducing the incidence of hyperventilation[16] A follow-up study showed that a medium sized adult bag (1100 ml) could provide a mean tidal volume of 623 ml ± 26 when used in conjunction with an intubating LMA[17] This produced a statistically significant difference when compared to the use of a conventional 1500 ml bag (741 ml ± 33). Other authors have demonstrated similar difficulties in achieving guideline consistent ventilations during CPR, with some minute volumes peaking at 21.3 litres[15]

In accordance with manufacturer specifications, the smaller 1000 ml capacity bag produces a maximum functional output of 750 ml – a characteristic that is likely to completely eliminate the incidence of overzealous volumes in excess of 1000 ml. With research suggesting that current capacity bags are likely to result in hyperventilation, we can also demonstrate an association to life-threatening secondary complications such as gastric insufflation, regurgitation, aspiration and barotrauma[18] While the effects of hypoxia and hypocapnia have proven to reduce the survivability of patients with severe head injury, the effect of suboptimal ventilation on outcomes for cardiac arrest patients are nowbeginning to demonstrate similar outcomes for swine models in cardiac arrest[19] It is now becoming more evident that "larger tidal volumes and ventilation rates can be associated with complications, whereas the detrimental effects observed with smaller tidal volumes appear to be acceptable."[4]

The results from this study have provided teaching staff with evidence to assist them in improving student ventilation during clinical simulation sessions. The findings from this study also highlight the need to investigate the ventilation ability of practicing Victorian paramedics.

This study is potentially limited due to its use of a mechanical lung model to depict the normal function and characteristic of a human lung under cardiac arrest conditions. Factors such as lower oesophageal sphincter pressure, peak airway pressure, peak airway flow and inspiratory time are all pertinent anomalies affecting ventilation accuracy in the setting of cardiac arrest. These factors were not investigated in this simulated model, and therefore consideration of these confounders must be taken before generalising results to human populations. Tidal volumes and ventilation rate were recorded using an analogue scale which requires accurate reading from a scale during the ventilation process. Therefore, human error in recording the value cannot be totally excluded.

## Conclusion

The delivery of optimal bag ventilation during CPR is often difficult even within the simplest of circumstances. Staggering degrees of suboptimal ventilation were observed for all three ventilation criteria with up to 97% of participants unable to achieve required tidal volumes when using a conventional adult 1600 ml self-inflating bag. We also demonstrated greater guideline consistent ventilation by introducing a smaller 1000 ml self-inflating bag. Suboptimal tidal and minute volumes fell by 27% and 23% respectively, with the introduction of a smaller capacity bag. These findings suggest that even the simplest of changes in operator equipment can potentially result in a greater efficacy of manual ventilation.

## Competing interests

The authors declare that they have no competing interests.

## Authors' contributions

ZN conceived the idea for the study. Both authors devised the study methodology and MB analysed the data. Both authors compiled the manuscript and both authors have read and approved the manuscript.

## Pre-publication history

The pre-publication history for this paper can be accessed here:



## References

[B1] Aufderheide TP, Sigurdsson G, Pirrallo R, Yannopoulos D, McKnite S, Briesen C, Sparks C, Conrad C, Provo T, Lurie KG (2004). Hyperventilation-induced hypotension during cardiopulmonary resuscitation. Circulation.

[B2] Pepe PE, Raedler C, Lurie KG, Wigginton JG (2003). Emergency ventilatory management in hemorrhagic states: elemental or detrimental?. J Trauma.

[B3] Davis DP, Dunford JV, Poste JC, Ochs M, Holbrook T, Fortlage D, Size MJ, Kennedy F, Hoyt DB (2004). The impact of hypoxia and hyperventilation on outcome after paramedic rapid sequence intubation of severely head-injured patients. J Trauma.

[B4] International Liaison Committee on R (2005). 2005 International Consensus on Cardiopulmonary Resuscitation and Emergency Cardiovascular Care Science with Treatment Recommendations. Part 2: Adult basic life support. Resuscitation.

[B5] Terndrup TE, Cherry RA, McCabe JB (1990). Comparison of ventilation performance: standard resuscitation bag and the resuscitation bag controller. J Emerg Med.

[B6] Walsh K, Loveday K, O'Rathaille M (2003). A comparison of the effectiveness of pre-hospital bag-valve-mask ventilation performed by Irish emergency medical technicians and anaesthetists working in a tertiary referral teaching hospital. Ir Med J.

[B7] Thomas SH, Orf J, Wedel SK, Conn AK (2002). Hyperventilation in traumatic brain injury patients: inconsistency between consensus guidelines and clinical practice. J Trauma.

[B8] Helm M, Hauke J, Lampl L (2002). A prospective study of the quality of pre-hospital emergency ventilation in patients with severe head injury. Br J Anaesth.

[B9] Augustine JA, Seidel DR, McCabe JB (1987). Ventilation performance using a self-inflating anesthesia bag: effect of operator characterisitics. Am J Emerg Med.

[B10] Martin PD, Cyna AM, Hunter WA, Henry J, Ramayya GP (1993). Training nursing staff in airway management for resuscitation. A clinical comparison of the facemask and laryngeal mask. Anaesthesia.

[B11] Helm M, Schuster R, Hauke J, Lampl L (2003). Tight control of prehospital ventilation by capnography in major trauma victims. Br J Anaesth.

[B12] Losert H, Sterz F, Kohler K, Sodeck G, Fleischhackl R, Eisenburger P, Kliegel A, Herkner H, Myklebust H, Nysaether J (2006). Quality of cardiopulmonary resuscitation among highly trained staff in an emergency department setting. Arch Intern Med.

[B13] Pitts S, Kellermann AL (2004). Hyperventilation during cardiac arrest. Lancet.

[B14] Milander MM, Hiscok PS, Sanders AB, Kern KB, Berg RA, Ewy GA (1995). Chest compression and ventilation rates during cardiopulmonary resuscitation: the effects of audible tone guidance. Acad Emerg Med.

[B15] O'Neill JF, Deakin CD (2007). Do we hyperventilate cardiac arrest patients?. Resuscitation.

[B16] Doerges V, Sauer C, Ocker H, Wenzel V, Schmucker P (1999). Smaller tidal volumes during cardiopulmonary resuscitation: comparison of adult and paediatric self-inflatable bags with three different ventilatory devices. Resuscitation.

[B17] Doerges V, Wenzel V, Neubert E, Schmucker P (2000). Emergency airway management by intensive care unit nurses with the intubating laryngeal mask airway and the laryngeal tube. Crit Care.

[B18] Hashimoto Y, Moriya F, Furumiya J (2007). Forensic aspects of complications resulting from cardiopulmonary resuscitation. Leg Med.

[B19] Idris AH, Wenzel V, Becker LB, Banner MJ, Orban DJ (1995). Does hypoxia or hypercarbia independently affect resuscitation from cardiac arrest?. Chest.

